# Naturalistic light exposure patterns in relation to medication status, mood symptoms, and chronotype

**DOI:** 10.1038/s44323-025-00062-0

**Published:** 2026-02-02

**Authors:** Rebecca J. Fitton, Julia E. Stone, Alicia C. Lander, Melisa Hrnjic, Malisa T. Burge, Elizabeth J. Buller, Amber P. White, Andrew J. K. Phillips, Sean W. Cain, Elise M. McGlashan

**Affiliations:** 1 School of Psychological Sciences and Turner Institute for Brain and Mental Health, Monash University, Melbourne, VIC Australia; 2 Melbourne School of Psychological Sciences, The University of Melbourne, Melbourne, VIC Australia; 3 Adelaide Institute for Sleep Health, Flinders Health and Medical Research Institute (Sleep Health), Flinders University, Adelaide, SA Australia; 4 Orygen, Parkville, VIC Australia

**Keywords:** Diseases, Health care, Neuroscience, Psychology, Psychology

## Abstract

Light assists in regulating mood, and selective serotonin reuptake inhibitors (SSRIs) increase non-visual light sensitivity. It remains unclear whether light exposure patterns differ between individuals taking SSRIs compared to unmedicated individuals with no psychiatric history, or how everyday light exposure relates to mood and chronotype. This study examined objective light exposure (melanopic EDI) in relation to medication status (SSRI *vs*. control), mood symptoms, and chronotype. Participants (*n* = 76; 38 SSRIs, 38 controls) completed at least one week of field light monitoring using a wearable sensor and questionnaires assessing mood (DASS-21) and chronotype (MEQ). Overall light exposure did not differ between groups. However, when accounting for group, greater morning light exposure was associated with lower depressive and stress symptoms, and more time spent above 50 melanopic EDI was associated with fewer depressive symptoms. Greater morningness was linked to higher morning and daytime melanopic EDI, more time in bright light (>50 and >250 melanopic EDI), and differences in light regularity. These findings show that light exposure, particularly its timing and amount, relates to mood and chronotype, regardless of SSRI use. Future research targeting light behaviour may offer an accessible, cost-effective strategy for improving mood in both clinical and non-clinical populations.

## Introduction

Light is the strongest environmental cue that synchronises circadian rhythms^1–3^, and light also acutely influences mood and alertness^1,4^. The effect of light on the circadian system depends on several factors, including the wavelength^5,6^, intensity^7^, timing and duration of exposure^8–10^, an individual’s prior light exposure history^11^, and their individual circadian light sensitivity^12,13^. Certain patient groups and psychiatric medications are associated with altered circadian light sensitivity^14–16^. Those experiencing depression exhibit reduced sensitivity during depressive episodes^15^, and acute (single) doses of Selective Serotonin-Reuptake Inhibitors (SSRIs), a popular class of antidepressant medication, profoundly increase the sensitivity of the circadian system to light^17^. Although the chronic influence of SSRIs on circadian light sensitivity has not yet been studied, an increase in light sensitivity may influence circadian function and mood in individuals undergoing antidepressant treatment.

How an SSRI-related increase in circadian light sensitivity impacts circadian regulation would depend on an individual’s light environment across the day. Even in the absence of medication, light exposure patterns play a key role in circadian rhythms and mood outcomes. Light exposure patterns that align with the natural light-dark cycle, such as bright daytime light and dim light/darkness at night, strengthen circadian signals^18,19^, assist in normalising circadian timing^20^, and are associated with lower risk of depression^21^ and improved mood outcomes^22^. In contrast, modern light patterns, such as bright evening light and less daytime light blur the day-night distinction^23^ and delay circadian phase^24^, resulting in disrupted and blunted circadian rhythms, commonly observed in mood disorders^25,26^. SSRIs may amplify the effects of light on mood and circadian rhythms. Healthy light patterns paired with an SSRI-induced increase in circadian light sensitivity may normalise the reduced circadian sensitivity to light seen in people experiencing depression^5^, possibly explaining the superior efficacy of combined SSRIs and bright light therapy^27–29^. However, SSRIs may also exacerbate circadian disruption linked to misaligned light patterns, contributing to the reduced treatment efficacy^30^ and higher depression symptoms in evening types compared to morning types^31^. Thus, everyday lighting environments may shape mood symptoms, particularly for those taking an SSRI.

Antidepressant efficacy is highly variable^32–34^, and everyday light behaviour may be predictive of mood symptoms, representing a promising target for intervention. Chronotype, an individual’s preference for the timing of daily activities, is associated with variable treatment responses^30^, and this may in part be driven by differences in habitual light exposure^35^. Identifying whether particular light exposure patterns are unique to certain populations or represent broader trends, and whether they relate to mood and chronotype across individuals, is critical in recognising modifiable environmental factors that contribute to mood disturbances. Ultimately, this knowledge could guide the development of tailored lighting strategies that complement pharmacological treatments, improve symptom management, and address the global rise of depression^36,37^.

In this study, we compared light exposure patterns between people taking SSRIs and controls, and examined how light exposure patterns relate to mood symptoms and chronotype (morning-evening preference). By clarifying these associations, we aim to inform future work on potential light-based strategies that could complement antidepressant treatment and support positive mood outcomes.

## Results

76 individuals (18-40 years, *M* = 25.03, *SD* = 4.24, 92.11% female) completed at least one week of field light-monitoring and questionnaires assessing mood (DASS-21) and chronotype (MEQ). The sample included 38 individuals regularly taking SSRIs and 38 matched unmedicated controls with no psychiatric history. Table 1 presents participant characteristics, including age, BMI, habitual sleep/wake timing, and self-reported depression, anxiety, and stress symptoms (DASS-21).

**Table 1:** Participant characteristics and questionnaire scores for the whole sample, control group, and SSRI group

	Whole sample *N* = 76		Controls *n* = 38		People taking an SSRI *n* = 38		Group comparison
	M(SD)	Range	M (SD)	Range	M (SD)	Range	*p*-value
Age (years)	25.03 (4.24)	18–38	23.90 (3.35)	18–30	26.16 (4.75)	18–38	**0.019**
BMI (kg m^-2^)	22.80 (3.63)	17.6–34.7	21.93 (2.68)	18.0–29.1	23.61 (4.24)	17.6–34.7	**0.036**
Bedtime (hh:mm)	00:06 (01:34)	21:22–03:55	00:21 (01:48)	21:22–03:23	23:51 (01:19)	22:01–03:55	0.167
Waketime (hh:mm)	08:36 (01:38)	05:56–14:16	08:21 (01:31)	05:56–14:16	08:50 (01:43)	06:04–14:01	0.201
MEQ	47.41 (9.66)	19–72	48.95 (9.38)	29–72	45.87 (9.82)	19–68	0.166
DASS-Depression	7.12 (5.97)	0–24	2.76 (2.91)	0–9	11.47 (4.99)	2–24	**<0.001**
DASS-Anxiety	4.92 (4.21)	0–18	2.66 (2.65)	0–8	7.18 (4.28)	1–18	**<0.001**
DASS-Stress	7.67 (5.11)	0–20	2.28 (3.18)	0–10	11.50 (3.56)	4–20	**<0.001**

Group comparisons were performed using independent samples *t*-tests. Times are given in hours and minutes. Average bedtime and wake time were determined from actigraphy data. *BMI* Body Mass Index, *MEQ* Morningness-Eveningness Questionnaire, *DASS* Depression Anxiety Stress Scales-21. Bolded results indicate statistically significant differences between groups (*p* < 0.05).

Across the sample, participants were exposed to an average of 678.38 melanopic EDI during the two hours following wake, 886.43 melanopic EDI during the day, and 35.64 melanopic EDI within the two hours before sleep onset. On average, participants spent 5.22 hours/day in light above 50 melanopic EDI and 2.05 hours/day in light above 250 melanopic EDI. Light Regularity Index averaged 80.09 at the median melanopic EDI threshold, and 84.75 at the 250 melanopic EDI threshold across the whole sample.

There were no significant group differences between those taking an SSRI and control participants in the light exposure metrics. Descriptive statistics for light metrics are shown in Table 2. Across all models, as expected, people taking SSRIs reported significantly higher levels of depression, anxiety, and stress symptoms compared to controls (all *p* < 0.001). Group remained a consistent and strong predictor in each model, independent of light exposure or sleep timing.

**Table 2:** Descriptive light exposure metrics and group comparisons

Variable	Whole sample	Control group	SSRI group	*p*-value
Morning light	678.38 (869.32), 1.76–4535.77	654.13 (849.58), 8.72–4535.77	704.02 (901.41), 1.76–3928.60	0.603
Daytime light	886.43 (858.21), 17.41–3865.63	980.65 (958.70), 47.27–3865.63	780.42 (729.55), 17.41 – 2999.04	0.680
Evening light	35.64 (47.29), 3.72–369.58	45.08 (62.15), 3.72–369.58	25.62 (19.39), 7.34–76.13	0.070
Time >50 mEDI	5.22 (1.90), 0.70–9.30	5.51 (1.77), 1.02–9.30	4.90 (2.01), 0.70– 8.56	0.179
Time >250 mEDI	2.05 (1.16), 0.05–5.04	2.19 (1.24), 0.27–5.04	1.91 (1.06), 0.05–4.42	0.325
LRI at median mEDI	80.09 (7.26), 62.10–97.54	78.49 (6.70), 63.23–90.89	81.98 (7.55), 62.10–97.54	0.056
LRI at 250 mEDI	84.75 (6.32), 72.48–99.47	84.23 (6.43), 72.48–96.85	85.36 (6.25), 76.68–99.47	0.484

All light metrics were calculated using melanopic EDI (mEDI). Group comparisons were performed using independent samples *t*-tests. Means (SD), with ranges in text. *LRI* Light Regularity Index, Morning, daily, and evening light were log_10_-transformed prior to testing but raw units are shown for interpretability. Times are given in hours and minutes. Sample sizes vary by model; see Table 6 for details.

### Morning, but not daytime or evening light, is associated with mood symptoms

Relationships between light exposure metrics and mood scores (DASS-21) across both the controls and individuals taking an SSRI are presented in Table 3.

**Table 3:** Linear regression models predicting DASS-21 symptoms from light exposure patterns

Light Predictor	Outcome	*B*	SE	β	t	*p*	R²
Morning light (0–2 hours post-wake)	Depression	−1.89	0.70	−0.23	−2.70	**0.009**	0.60
	Anxiety	−1.84	0.64	−0.20	−1.84	0.070	0.34
	Stress	-1.50	0.59	−0.21	−2.53	**0.014**	0.62
Daytime light	Depression	−0.31	1.04	−0.03	−0.30	0.764	0.55
	Anxiety	0.99	0.94	0.11	1.05	0.296	0.31
	Stress	0.58	0.82	0.05	0.70	0.484	0.62
Evening light (0–2 hours pre-sleep onset)	Depression	1.31	1.33	0.09	0.98	0.329	0.54
	Anxiety	0.80	1.31	0.07	0.61	0.544	0.25
	Stress	0.05	1.24	0.04	0.04	0.967	0.53
Time > 50 mEDI	Depression	−0.52	0.25	−0.17	−2.06	**0.043**	0.57
	Anxiety	−0.26	0.23	−0.12	−1.13	0.262	0.32
	Stress	−0.16	0.21	−0.06	−0.73	0.466	0.61
Time > 250 mEDI	Depression	−0.18	0.43	−0.04	−0.43	0.673	0.55
	Anxiety	0.20	0.38	0.05	0.51	0.610	0.31
	Stress	−0.01	0.35	−0.01	−0.02	0.981	0.61
LRI at median mEDI	Depression	0.03	0.06	0.04	0.45	0.654	0.57
	Anxiety	0.10	0.06	0.17	1.55	0.125	0.31
	Stress	0.02	0.06	0.03	0.41	0.685	0.61
LRI at 250 mEDI	Depression	0.06	0.07	−0.07	−0.08	0.936	0.57
	Anxiety	−0.05	0.07	−0.07	−0.67	0.504	0.29
	Stress	−0.06	0.06	−0.08	−0.08	0.321	0.61

All light metrics are calculated using melanopic EDI (mEDI). Each row represents a separate linear regression model with one light exposure variable as the predictor. All models adjusted for group (SSRI *vs*. control); morning light models also included waketime, and evening light models included sleep onset time. Sample sizes vary by model; see Table 6 for details. Bolded results indicate statistically significant associations (*p* < 0.05). *β* = standardized regression coefficient; *SE* standard error of the coefficient, *LRI* Light Regularity Index.

**Fig. 1:**
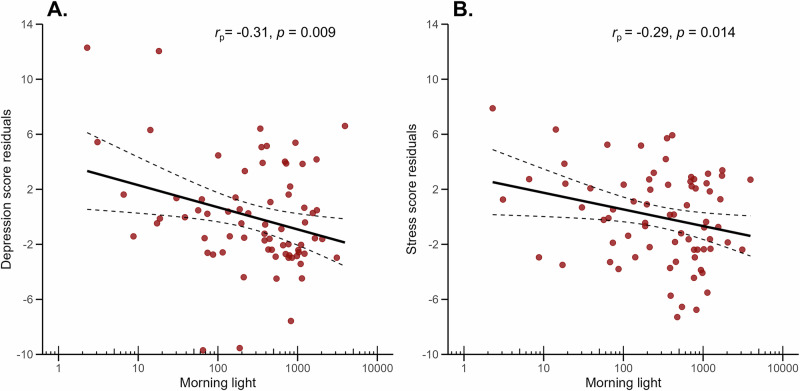
Associations between morning light exposure and depressive and stress symptoms. **A** Depression symptom residuals and **B** stress symptom residuals are plotted against morning light exposure (2 hours post-wake, melanopic EDI; log_10_-transformed x-axis). Residual scores were obtained from models adjusting for group and wake time, representing the variance in the outcome variable after accounting for covariates. Solid black lines represent linear regression fits; dashed lines indicate 95% confidence intervals. In both panels, greater morning light exposure was significantly associated with lower symptom residuals. Corresponding partial correlation coefficients (*r*_p_*)* and two-tailed *p*-values (*p*) displayed within each panel reflect the association between the residualized outcome variable and predictor.

When accounting for group and waketime, greater exposure to morning light (0–2 hours post-wake) was significantly associated with less severe depressive symptoms (*B* = −1.89, *p* = 0.009) and fewer stress symptoms (*B* = −1.50, *p* = 0.014), as shown in Fig. 1. Earlier waketimes were also associated with higher symptom scores for anxiety (*B* = −0.59, *p* = 0.040), and stress (*B* = −0.69, *p* = 0.010), but not depression scores.

Neither daytime light exposure nor evening light exposure were significantly associated with depression, anxiety, or stress symptoms. Sleep onset time, included as a covariate in the evening light models, was also not a significant predictor.

### Time spent in light and mood symptoms

**Fig. 2:**
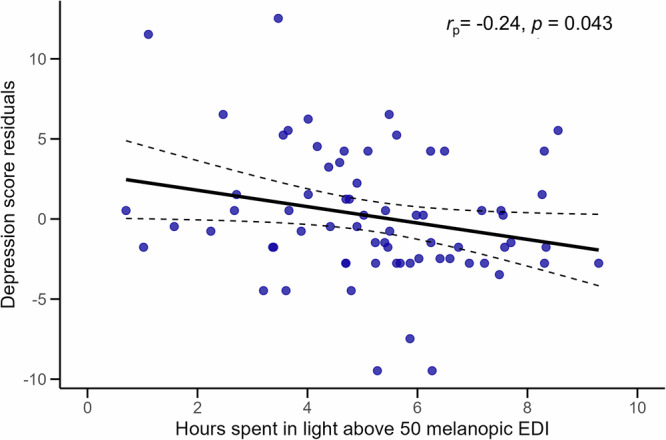
Greater time spent in light above 50 melanopic EDI is associated with lower depressive symptoms residuals. Scatterplot depicting the relationship between hours spent above 50 melanopic EDI (*x*-axis) and depression score residuals (*y*-axis). Depression scores have been residualized to remove the effects of group, such that residuals represent the variance in depression scores unexplained by group. The solid black line shows the linear regression fit of time spent in light on residual scores, with dashed lines representing 95% confidence intervals. Corresponding partial correlation coefficients (*r*_p_) and two-tailed *p*-values (p) shown reflect the association between the residualized outcome variable and predictor.

Amount of time spent above 50 melanopic EDI each day was significantly associated with lower depressive symptoms (*B* = −0.52, *p* = .043; see Fig. 2), but not with anxiety or stress scores. In contrast, amount of time spent above 250 melanopic EDI each day was not significantly associated with any mood symptom outcomes.

### Associations between light exposure patterns and chronotype

Associations between each light exposure metric and chronotype (MEQ) are presented in Table 4.

**Table 4:** Associations between light exposure variables and MEQ score (chronotype), controlling for group

Light Predictor	*B*	SE	β	t	*p*	R²
Morning light	4.13	1.52	0.31	2.72	**0.008**	0.13
Daytime light	10.28	2.20	0.49	4.68	**<0.001**	0.28
Evening light	-6.07	3.69	−0.20	−1.64	0.105	0.08
Time > 50 mEDI	2.19	0.57	0.42	3.83	**<0.001**	0.21
Time > 250 mEDI	3.82	0.92	0.45	4.13	**<0.001**	0.23
LRI at median mEDI	0.66	0.15	0.51	4.51	**<0.001**	0.28
LRI at 250 mEDI	−0.71	0.17	−0.48	−4.29	**<0.001**	0.27

All light metrics were calculated using melanopic EDI (mEDI). Each model includes one light variable as the predictor and the MEQ score as the outcome, with group (SSRI *vs*. control) included as a covariate. Sample sizes vary by model; see Table 6 for details. Group was not a significant covariate for any model. *LRI* Light Regularity Index.

**Fig. 3:**
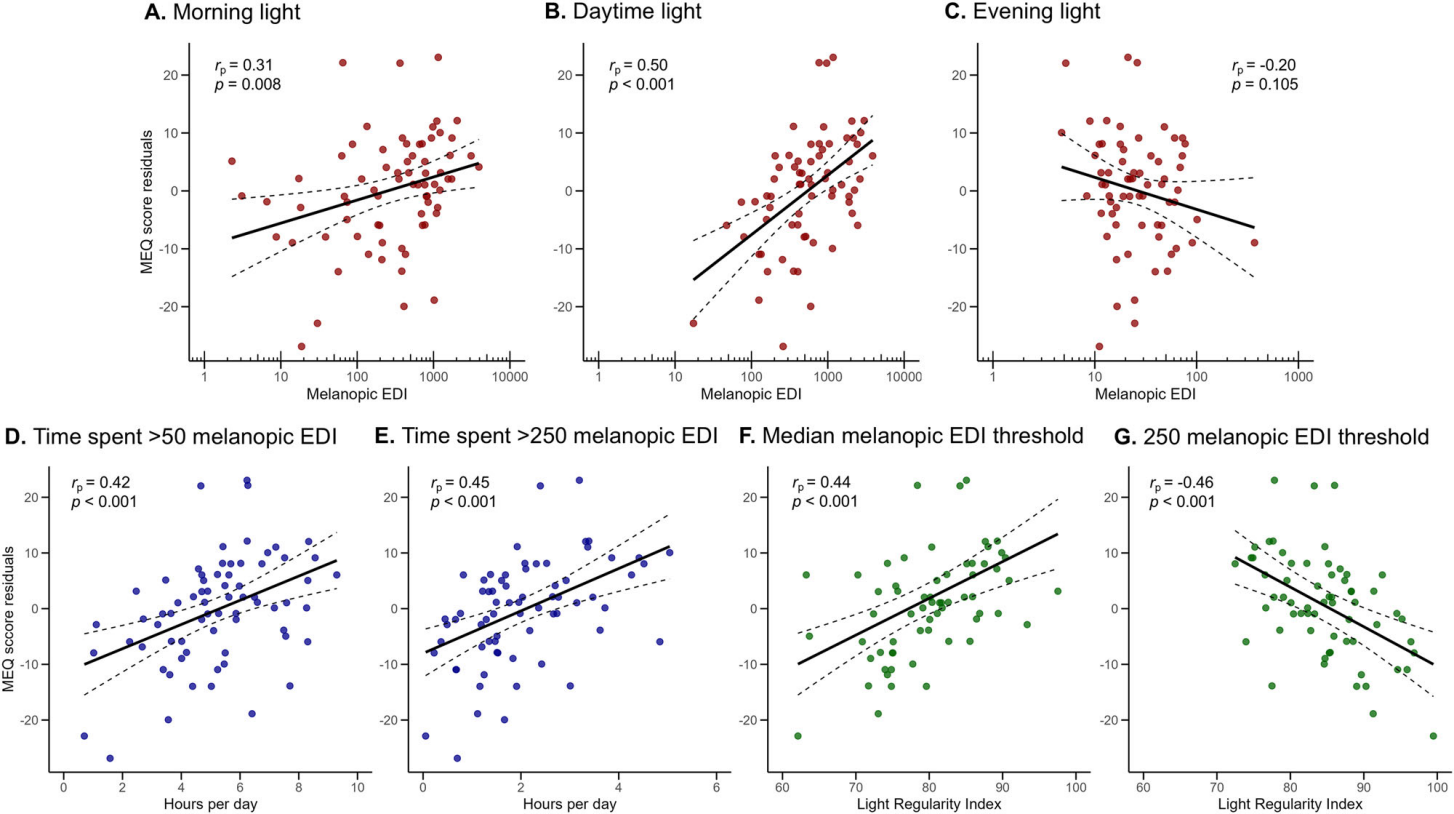
Associations between light exposure patterns and chronotype residuals. Each panel shows a scatterplot of the associations between residual Morning-Eveningness Questionnaire (MEQ) scores (*y*-axis) and different melanopic EDI light metrics (*x*-axis): morning light (**A**), daytime light (**B**), evening light (**C**), time spent above 50 melanopic EDI (**D**), time spent above 250 melanopic EDI (**E**), Light Regularity Index at median melanopic EDI threshold (**F**), and Light Regularity Index at 250 melanopic EDI threshold (**G**). Residuals were derived from models adjusting for group and represent the variance in MEQ after accounting for group. Each dot represents one participant. Solid black lines indicate linear regression fits; dashed lines indicate 95% confidence intervals. Log-transforms were applied to the *x*-axis of **A**–**C** to match regression model specifications. All associations were statistically significant except that between evening light and chronotype. Corresponding partial correlation coefficients (*r*_p_) and two tailed *p*-values (*p*) are annotated within each panel reflect the associations between each predictor and the residualized outcome variable.

Greater morning light exposure was significantly associated with higher MEQ scores (*B* = 4.13, *p* = 0.008), indicating a greater tendency toward morningness. Similarly, individuals exposed to more daytime light (*B* = 10.28, *p* < 0.001), more time above 50 melanopic EDI (*B* = 2.19, *p* < 0.001), and more time above 250 melanopic EDI (*B* = 3.82, *p* < 0.001) reported significantly higher MEQ scores. Evening light exposure was not significantly associated with MEQ score (*B* = -6.07, *p* = 0.105). Greater light regularity at median melanopic EDI threshold was significantly associated with higher MEQ scores, indicating greater morningness (*B* = 0.66, *p* < 0.001). In contrast, individuals with greater light regularity at 250 melanopic EDI threshold reported lower MEQ scores (*B* = −0.71, *p* < 0.001), demonstrating that individuals with more regular light patterns reported greater eveningness. Visual representations of these associations are shown in Fig. 3.

## Discussion

In this study, we examined how objective melanopic light exposure patterns related to mood symptoms and chronotype under free-living conditions in individuals taking SSRIs and matched controls. Our findings show that, although overall light exposure patterns did not differ between groups, greater exposure to bright morning light and spending more time in light above dim-moderate light levels (50 melanopic EDI) independently predict fewer depressive symptoms across the whole sample. Furthermore, morning-types exhibited greater light exposure in the morning and daytime, spent more time in light exceeding both low and bright thresholds (50 melanopic EDI and 250 melanopic EDI), and demonstrated differences in regularity of light exposure at both median and 250 melanopic EDI thresholds compared to evening-types. These findings indicate that light behaviour plays an important role in mood regulation, regardless of medication status, and that morning-evening preference relates to differences in daily light exposure patterns.

Brighter melanopic light in the first two hours after waking was associated with fewer depressive and stress symptoms across our sample. Bright morning light may influence mood through multiple pathways. Morning light exposure can advance circadian timing^38–40^, counteracting the delaying impact of evening light that is often linked to low mood and depressive symptoms^24^. Brighter mornings may also strengthen the amplitude of circadian rhythms^41^, which are typically blunted in depressive disorders^25,26^. Light information is also received in brain areas related to mood regulation, such as the amygdala and habenula^4,42,43^. As a result, light acutely improves mood and has been shown to impact self-perception (*i.e*., how an individual may think about themselves)^44^. Therefore, greater levels of morning light are likely beneficial for mood through both circadian and non-circadian mechanisms of the non-visual effects of light. Notably, the average morning light levels in our sample were significantly below typical therapeutic thresholds used in clinical light therapy^45^, indicating that even low-to-moderate levels of morning light may be beneficial for mood. This suggests potential for adjunctive naturalistic light interventions, such as spending time outside, which may be more accessible than clinical light therapy, to be beneficial in both patients and the community in general. Future randomized trials are needed to determine whether changing everyday lighting behaviours can effectively improve mood symptom severity in clinical and non-clinical populations.

We found that spending more time across the day in light above 50 melanopic EDI was associated with fewer depressive symptoms. This suggests that simply avoiding prolonged time in dim light environments may be an important, yet largely overlooked, factor in supporting mood. By contrast, the amount of time spent in light above 250 melanopic EDI, the recommended minimum melanopic EDI during the day^46^, was not associated with mood symptoms. This may in part reflect the fact that modern lighting environments do not sufficiently reach these levels^47–50^. Both groups spent an average of ~2 hours above 250 melanopic EDI, which is lower than durations previously associated with lower odds of depressive symptoms^51^, and therefore may not be sufficient to influence mood outcomes. Alternatively, the association may be influenced less by the duration of exposure above 250 melanopic EDI and more by whether individuals receive any exposure above this threshold. The distinction between the amount of time spent above the threshold and simply receiving any exposure above it is difficult to clarify without longitudinal data examining how changes in light exposure relate to mood over time. The lower threshold of 50 melanopic EDI may better capture variability between participants in terms of extended time spent in relative ‘darkness’, throughout the day. Together, our findings highlight the importance of daily light exposure above dim-moderate conditions. However, it remains unclear whether reduced light exposure across the day precedes depressive symptoms or results from mood-related changes in behaviour.

Regardless of whether changes in light behaviour precede or follow the onset of mood symptoms, spending more time in dimmer lighting conditions may contribute to mood disturbances. Low daytime light exposure has been associated with the onset of depressive episodes in seasonal affective disorder^52^, atypical depressive symptoms in non-clinical groups^47^, and greater depression risk in population-based studies^53^. Insufficient time spent in light may contribute to blunted circadian amplitude^25^ and reduce activation of intrinsically photosensitive retinal ganglion cells (ipRGCs)^15,54,55^, which mediate direct mood and alertness effects via projections to mood and alertness-regulating brain areas^4,42,43^. These pathways provide a link between low light exposure and depressed mood. Prolonged time spent in dim environments may also reflect or reinforce sedentary or socially withdrawn lifestyles, further exacerbating symptoms. Thus, low light environments may both signal and sustain poor mood, underscoring the importance of promoting brighter daytime environments as a potential preventative strategy.

Prior studies have shown that less outdoor light exposure relates to antidepressant use^51^ and that greater amounts of evening light increase depression risk^21^. However, we did not observe any significant differences in light exposure patterns between individuals taking an SSRI and controls. Our results may be explained by clinical heterogeneity in our sample. Our sample included some people taking SSRIs reporting minimal to mild depressive symptoms, and some controls reporting moderate depressive symptoms according to the DASS-subscale scores. This overlap in symptom severity across groups may have reduced the contrast in light exposure patterns previously seen clinical and non-clinical populations^56^, masking group-level differences. Importantly, serotonin agonists and SSRIs are known to alter circadian light sensitivity in nocturnal animals^57–59^, diurnal animals^60^, and humans^17^, suggesting that similar light environments could impact mood differently in medicated versus unmedicated individuals. SSRI-induced heightened circadian light sensitivity could lead to greater disruptions or benefits from daily light exposure patterns compared to controls, even when overall light exposure patterns appear overtly similar. For example, bright light therapy tends to be more effective when combined with SSRIs than when used alone^28^, highlighting the interaction between medication and light environments. Longitudinal studies examining changes in light exposure and circadian light sensitivity after starting antidepressant treatment are essential for understanding how SSRIs influence the mood-enhancing or mood-disrupting effects of everyday lighting in the context of individual-level circadian light sensitivity and light exposure.

Both groups had evening melanopic EDI well above the recommended 10 melanopic EDI threshold in the two hours before sleep^46^. This may reflect lifestyle factors, as a large proportion of the sample were university students (69.7%), particularly in the control group (81.6%), which may contribute to differing routines and exposure to evening light. For example, students often spend evenings studying or socialising, and are more likely to work part-time evening jobs, potentially increasing exposure to artificial light at night.

Evening light is known to disrupt sleep and circadian rhythms, and can predict psychiatric disorders^13,21,23,61^. However, we did not observe associations between evening light and mood symptoms in this sample. This may be in part driven by high overall evening light levels, which exceeded the recommended evening light exposure levels, meaning we had relatively few participants with ‘ideal’ evening light exposure. Additionally, previous studies have shown a > 50-fold difference in response to evening light among young adults^12^, and this variability is likely amplified in a sample including those taking medications like SSRIs^17^. The association between evening light and mood may depend on individual sensitivity to light, with the same evening light environment resulting in more adverse effects for people with greater sensitivity. This may be particularly relevant here, given our observed mean evening light exposure (35.64 melanopic EDI) is close to the mean light level required to suppress melatonin by 50% during the evening in young adults^12^. This level of evening light exposure would therefore amplify the impact of inter-individual differences in circadian light sensitivity. Together, differences in non-visual light sensitivity and uniformly high exposure may have masked relationships between evening light and mood.

We observed that greater morningness was associated with increased morning and daytime light, greater time spent in light, and differences in light regularity depending on the threshold used. We found that evening types exhibited more regular exposure to light using the 250 melanopic EDI threshold, but less regular exposure when regularity was assessed using each individual’s median melanopic EDI threshold. Regularity at a fixed 250 melanopic EDI threshold may reflect evening types consistent but lower light levels across the waking day, compared to morning types. Morning types tend to wake and start their day earlier, coinciding with their peak physical and cognitive performance in the early part of their day^62^, which may encourage seeking more bright morning and daytime light^63^. In contrast, evening types often wake later and reach peak performance closer to bedtime, leading to less morning light, lower overall light exposure, and greater irregularity in their median daily light patterns. Notably, all of our time-based light metrics (*i.e*., morning, daytime, and evening light) were calculated relative to an individual’s sleep-wake timing on a given day, rather than with static clock times (*e.g*., 0700–0900). As a result, the differences in light exposure between morning and evening types are not solely due to morning types waking earlier, and therefore receiving more light in the “morning”. People with greater eveningness were exposed to less light above 50 melanopic EDI, suggesting that chronotype may affect how much light people seek out or avoid. It remains unclear whether these light exposure differences are driven by intrinsic chronotype traits that influence the propensity to seek light, or whether altered light exposure patterns themselves contribute to shaping morning-eveningness. Indeed, evidence from camping studies shows that when individuals are exposed to natural light-dark cycles without artificial light, variability in sleep and circadian timing decreases^64^, indicating that environmental light may play a role in producing differences in morning-evening preference. Differences in light regularity patterns between morningness and eveningness may contribute to the known greater variability in circadian timing, increased sleep disturbances, and higher rates of mood symptoms in evening types^31,65–68^. Irregular light input to the circadian system, relative to an individual’s median light exposure, may underlie some of these adverse outcomes. Future longitudinal and experimental research is needed to clarify these causal relationships and determine whether modifying light exposure can improve circadian regularity and mood, especially in evening types.

It is important to note that our sample was predominantly women, which likely reflects broader trends with women being twice as likely to experience depression^69^, and more likely to participate in mental health research. The sex imbalance may limit generalisability of our findings across sexes. Women tend to be more sensitive to bright light^70^, have higher rates of depression^69^, and respond differently to SSRI treatment^71^. It is possible that sex moderates the relationships between light exposure patterns and antidepressant treatment or mood outcomes more broadly. Further research should aim for a more balanced sex representation and directly examine the potential interacting effects of sex on associations between light exposure, antidepressant treatment, and mood.

This study provides novel insights into objectively measured naturalistic light exposure patterns in relation to medication status, mood symptoms, and chronotype. While we found no differences in overall light exposure between those taking an SSRI and controls, greater morning melanopic light exposure independently associated with fewer depressive and stress symptoms, and longer cumulative time spent above 50 melanopic EDI was associated with fewer depressive symptoms. Additionally, brighter, more regular, and longer time spent in moderate or bright light were associated with morningness. These findings underscore the potential value of incorporating targeted field-based approaches, such as increasing morning light exposure and increasing the amount of time in at least moderate light, into interventions aimed at supporting mood in both medicated and unmedicated populations.

## Methods

Ethical approval was obtained from the Monash University Human Research Ethics Committee (Project IDs: 22905, 20590, 31958). Participants provided written informed consent and were reimbursed for their time. All procedures were conducted in accordance with the Declaration of Helsinki.

### Participants

A total of 76 participants aged between 18 and 40 years (*M* = 25.03, *SD* = 4.24) were recruited through the general community using social media advertisements (*e.g*., paid Facebook ads, posts in relevant Facebook groups), physical flyers at nearby university campuses and at general practitioners, and a pool of past participants that provided consented to future contact. All participants completed at least one week of field monitoring of sleep, mood, and light exposure patterns between 2021 and 2024. Half of these participants (*n* = 38) were taking a selective serotonin reuptake inhibitor (SSRI) for at least 8 weeks (44.7% escitalopram, 34.2% sertraline, 13.2% fluoxetine, and 7.9% citalopram) and self-reported a psychiatric history (42.1% depression, 5.3% of anxiety, and 52.6% both depression and anxiety). The control group (*n* = 38) was selected from a database of participants to be matched to the SSRI group for sex (92.11% female, 7.89% male) and age (within two years on a per participant basis) ensuring comparability with the clinical group. Controls had no self-reported psychiatric history and were not taking psychiatric medication.

Participants completed a range of online and over-the-phone questionnaires to assess preliminary eligibility, including the Morning Eveningness Questionnaire (MEQ)^72^ to measure chronotype. Participants were excluded if they had a self-reported medical condition, were taking medications (except an SSRI for the patient group), worked shift work in the past three months, had travelled across time zones in the past 3 months, or if they were at high risk of suicide, as indicated by a score of 2 or 3 on item 9 of the Beck Depression Inventory-II^73^.

Data collection occurred across all seasons, with most participants studied during winter/autumn (68.4% of the controls and 57.9% of the SSRI group). The study catchment area spanned approximately 36.77°S to 38.54°S in Victoria, Australia.

### Field monitoring

Field sleep, light, and mood data were collected for at least one week in Victoria, Australia. All participants were instructed to go about their usual activities while collecting data. Participants were asked to wear an actigraphy device (Philips Actiwatch Pro, Philips Respironics, PA, USA, or ActivInsights GENEActiv, Cambridgeshire, UK) on their non-dominant wrist to record activity in one-minute intervals. They were instructed to wear the device during both wake and sleep periods, removing it only when it could be damaged (*e.g*., during showers or high-impact sports). Instances of device removal were documented in a daily sleep diary completed via the MyCap App (REDCap, Vanderbilt University, Nashville, TN, USA). Participants also reported daily sleep and wake times in the electronic sleep diary.

To assess light exposure, participants wore a prototype device similar to that described in Cain et al. (2020)^23^, around the neck on a lanyard to capture light exposure during waking periods. This device features an array of light sensors (spectral range 410 – 760 nm), allowing for the detailed recording of light environments in two-minute intervals, and provides calculated epoch-level measures of melanopic effective daylight illuminance (EDI). Participants were advised to remove the pendant only when it risked damage, and to charge it each night. Participants were asked to position the light sensor upwards to monitor light exposure during sleep periods. Participants completed the Depression Anxiety Stress Scales-21 (DASS-21;^74^) online within one week of field monitoring to assess self-reported symptoms related to depression, anxiety, and stress over the past week. The 21-item scale uses a 4-point Likert scale (0–3), with higher scores indicating greater levels of psychological distress across three 7-item subscales (depression, anxiety, and stress).

### Light and sleep data processing

Sleep and wake intervals were determined using visual inspection of algorithm-determined sleep in Actiware for Philips Actiwatches (*n* = 44), with adjustments based on participants’ daily sleep diaries. For participants using GENEActiv devices (*n* = 32), sleep data were cleaned in R using the GGIR package (version 3.2-6;^75^). The HDCZA algorithm was used to detect the sleep period based on patterns of low movement, using predefined thresholds for wrist angle change (5 degrees), and duration (5 minutes), while ignoring non-wear periods. In instances of missing sleep data, sleep/wake times were manually extracted from sleep diaries (1.59% of nights in Actiwatch users, 7.59% in GENEActiv users).

Data were then processed in MATLAB R2022b using a custom script. Sleep data were converted into a binary minute-by-minute vector (1 = awake, 0 = asleep), aligned to calendar dates, and corrected for midnight crossings. No nights of sleep data were missing. Light exposure data from the prototype device were timestamped and aligned to the sleep-wake vector. This included melanopic EDI values, which were used to calculate all subsequent light exposure metrics. Melanopic EDI was used as it is known to provide the most accurate measure of light’s impact on the human circadian system^55^. This metric reflects the sensitivity of intrinsically photosensitive retinal ganglion cells (ipRGCs), which are responsible for non-visual responses such as melatonin suppression and circadian phase shifting^76–78^. Extremely high light values (>1,000,000) that exceeded natural sunlight were excluded due to device error (*M* = 0.02%, *SD* = 0.07%).

Light data were cleaned in three steps to remove invalid or implausible values, resulting in an average of 19.2% of light data removed per participant. See Table 5 for details of the light data cleaning procedure.

**Table 5:** Light data cleaning procedures

Rule	Description	Mean % Removed (SD)	Range (%)
1	Coverage of light sensor: values < 1 melanopic EDI during wake	13.63 (7.67)	1.90–48.14
2	Sudden drops (<10 melanopic EDI surrounded by >10 melanopic EDI values)	1.67 (1.43)	0–6.11
3	Flatness: very low variability during wake (<0.05 SD over 10 min), indicating the sensor may have been stationary (unworn) or covered	3.87 (4.77)	0–25.64
	Total cleaned	19.17 (13.87)	0–48.14

Each rule was applied sequentially. Cleaned and raw light data were visualised daily and checked against sleep intervals for each participant to check for abnormalities or misalignment prior to the calculation of metrics.

### Light exposure metrics

Melanopic light exposure metrics were calculated using cleaned, timestamped light and sleep/wake data. Sleep onset and wake times were derived from binary sleep data to align light exposure windows across days. The following light metrics were defined to measure different aspects of light exposure patterns:

(i) Morning light: This was calculated as the average melanopic EDI within the 2-hour window after wake onset, calculated daily, and then averaged across days.
(ii) Daytime light: This was calculated as the average melanopic EDI between the end of the 3-hour post-wake window and the start of the 3-hour pre-sleep window.
(iii) Evening light: This was calculated as the average melanopic EDI within 2 hours before sleep onset, calculated daily, and then averaged across days.
(iv) Total daily hours spent above 50/250 melanopic EDI: This was calculated as the total duration spent above the thresholds of 50 and 250 melanopic EDI. The 50 mEDI threshold represents typical indoor lighting conditions and the upper bound of “dim” conditions used in prior light exposure studies, where alerting effects are typically not observed below this level^43^. The 250 mEDI threshold corresponds to the recommended minimum daily light exposure to support circadian health^46^.
(v) Light Regularity Index (LRI)^79^: This was calculated at thresholds of the median melanopic EDI for each individual and 250 melanopic EDI, where the percentage probability of an individual being in the same light environment (*e.g*., light above 250 melanopic EDI *vs*. light below 250 melanopic EDI) at any two time-points 24 hours apart was calculated and averaged across the study. Scores are then scaled to a range of 0-100, with higher values indicating greater regularity in light exposure.

For time-based metrics, only windows with at least 70% valid data were retained (*e.g*., 70% of the data within the 2-hour post-wake window was required to calculate the metric for a given day). LRI threshold calculations required a total of three days of valid epochs and a minimum of 50% valid epoch comparisons between any two time-points 24-hours apart. Daily light exposure and time spent above light thresholds required at least 8 hours of valid light data for calculation (valid recording days: *M* = 5.48 days, *SD* = 1.66).

### Data preparation

All light exposure variables were winsorized at the 5th and 95th percentiles to reduce the influence of extreme outliers. The percentage of values adjusted per variable ranged from 10.0% to 10.7%. Morning light, daytime light, and evening light variables were then log_10_-transformed to reduce skewness and improve normality of residuals, as they each had positive skew. Time spent above threshold variables and light regularity thresholds were close to normally distributed and analysed without transformation. Each analysis included only those participants with at least three valid days of data for that specific light metric. As a result, the sample size varied slightly across analyses depending on data completeness for each light metric (range: *n* = 63–70). In all cases, group sizes remained similar and relatively equal (see Table 6).

**Table 6:** Final sample size per light exposure metric after data cleaning and exclusion

Light metric	Total *n*	Controls	SSRI
Morning light	66	34	32
Daytime light	68	36	32
Evening light	66	34	32
Time spent > 50 mEDI	70	36	34
Time spent > 250 mEDI	70	36	34
LRI at median mEDI	63	34	29
LRI at 250 mEDI	63	34	29

All light metrics are calculated using melanopic EDI (mEDI). *LRI* Light Regularity Index.

After preprocessing, all light exposure metrics (except LRI thresholds, which were calculated at participant level) were averaged across valid days to generate one value per participant per variable.

### Data analysis

All statistical analyses were conducted in RStudio (version 4.5.0). Descriptive statistics were calculated for light exposure variables and reported in raw units for interpretability. Independent samples *t*-tests were used to compare light exposure metrics between the SSRI and control groups. Associations between light exposure and mood symptoms or chronotype were examined using linear regression models via the lm() function in RStudio. All tests were two-tailed with an alpha level of 0.05. Visualisations were produced using the ggplot2 package.

Linear regression models were used to investigate associations between light exposure metrics and both mood symptoms and chronotype across both the control sample and people taking an SSRI. For mood symptoms (depression, anxiety, or stress symptoms from the DASS-21), separate models were constructed for each light exposure metric. All models included group (SSRI *vs*. control) as a covariate. Waketime was included as an additional covariate in morning light models, and sleep onset time was included in evening light models to account for variability in sleep timing. To examine associations between light exposure metrics and chronotype, separate linear regressions were conducted for each light metric, with group included as a covariate. Sleep timing variables were not included in these models due to conceptual overlap with chronotype, which relates to individual preferences for sleep and wake timing.

## Data Availability

Raw data is available from the corresponding author upon request.

## Code availability

Data analysis code is available upon reasonable request.
